# Health Internet Technology for Chronic Conditions: Review of Diabetes Management Apps

**DOI:** 10.2196/17431

**Published:** 2021-08-31

**Authors:** Sweta Sneha, Srivarun Thalla, Ishaan Rischie, Hossain Shahriar

**Affiliations:** 1 Kennesaw State University Kennesaw, GA United States; 2 Rice University Houston, TX United States

**Keywords:** chronic disease, diabetes, blood glucose, diabetes management, mHealth disease apps, diabetes apps, best Apple diabetes apps

## Abstract

**Background:**

Mobile health (mHealth) smartphone apps have shown promise in the self-management of chronic disease. In today’s oversaturated health app market, selection criteria that consumers are employing to choose mHealth apps for disease self-management are of paramount importance. App quality is critical in monitoring disease controls but is often linked to consumer popularity rather than clinical recommendations of effectiveness in disease management. Management of key disease variances can be performed through these apps to increase patient engagement in disease self-management. This paper provides a comprehensive review of features found in mHealth apps frequently used in the self- management of diabetes.

**Objective:**

The purpose of this study was to review features of frequently used and high consumer-rated mHealth apps used in the self-management of diabetes. This study aimed to highlight key features of consumer-favored mHealth apps used in the self-management of diabetes.

**Methods:**

A 2-fold approach was adopted involving the Apple iOS store and the Google search engine. The primary search was conducted on the Apple iOS store using the term “diabetes apps” (device used: Apple iPad). The top 5 most frequently used mHealth apps were identified and rated by the number of consumer reviews, app ratings, and the presence of key diabetes management features, such as dietary blood glucose, A_1C_, insulin, physical activity, and prescription medication. A subsequent Google search was conducted using the search term “best Apple diabetes apps.” The top 3 search results—“Healthline,” “Everyday Health,” and “Diabetes Apps–American Diabetes Association”—were explored.

**Results:**

In total, 12 mHealth apps were reviewed due to their appearing across 4 evaluated sources. Only 1 health app—Glucose Buddy Diabetes Tracker—appeared as the most frequently used within the Apple iOS store and across the other 3 sources. The OneTouch Reveal app ranked first on the list in the iOS store with 39,000 consumer reviews and a rating of 4.7 out of 5.0 stars but only appeared in 1 of the other 3 sources. Blood glucose tracking was present across all apps, but other disease management features varied in type with at least 3 of the 5 key features being present across the 12 reviewed apps. Subscription cost and integration needs were present in the apps which could impact consumers’ decision to select apps. Although mobile app preference was assessed and defined by the number of consumer reviews and star ratings, there were no scientific standards used in the selection and ranking of the health apps within this study.

**Conclusions:**

mHealth apps have shown promise in chronic disease management, but a surge in development of these nonregulated health solutions points to a need for regulation, standardization, and quality control. A governing body of health IT professionals, clinicians, policymakers, payors, and patients could be beneficial in defining health app standards for effective chronic disease management. Variabilities in features, cost, and other aspects of management could be reduced by regulatory uniformity, which would increase patient engagement and improve disease outcomes.

## Introduction

### Background

Health information technology (HIT) has been touted as a solution for prolonged management of chronic diseases, such as diabetes, and their associated lifestyle fluctuations in diet, exercise, and prescription medication. Remote monitoring of diabetes through mobile health (mHealth) apps is one of the early management mediators of the disease and has increased in use throughout recent years [1]. Over 3500 apps and counting have been designed and tailored specifically for chronic conditions [2]. Many of these apps include mobile developments aimed at effectively addressing disease management through increased patient self-management to reduce associated health care costs. The effective application of information technology in chronic disease management holds the potential to significantly impact health care outcomes through facilitated treatment adherence which integrates both compliance and modifiable health behaviors (ie, diet and exercise) [3]. Improving adherence through the use of HIT has become a critical focus within the health care industry to improve patient outcomes and reduce associated health care costs. Due to the need for continuous monitoring and long-term care of chronic disease sufferers, early HIT advancements, such as telehealth and telemonitoring, have been employed to reinforce patient adherence to chronic disease controls [4]. HIT solutions have continued to evolve as mHealth technologies have become popular platforms for delivery of at-home care and have developed new capabilities that enable patients to self-manage chronic conditions.

**Figure 1:**
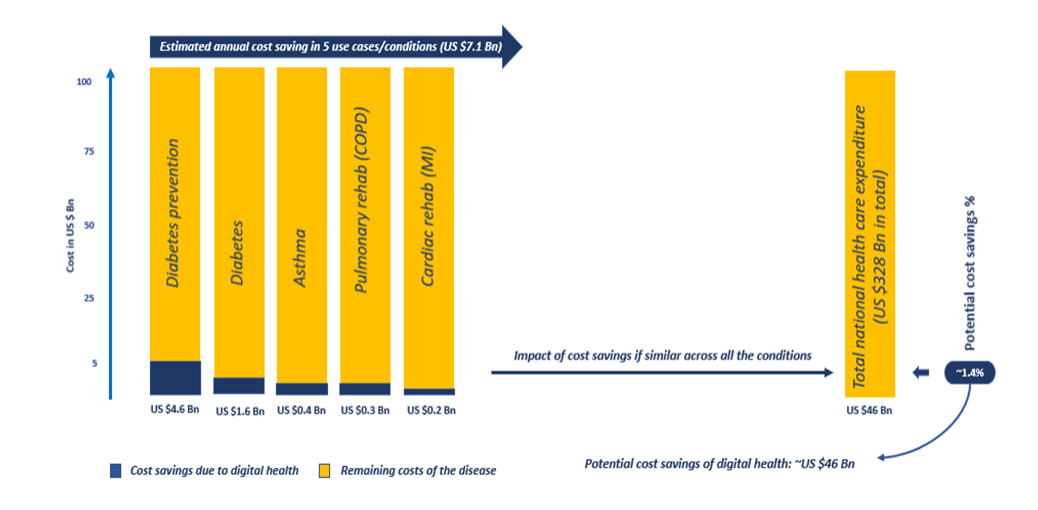
Potential annual cost savings with the use of digital health apps [5]. Bn: billion; COPD: chronic obstructive pulmonary disease; MI: myocardial infarction. (Adopted from Aitken et al, IQVIA, 2017.)

Health-related smartphone apps provide a “platform for the delivery of self-management interventions that are highly adaptable, have low healthcare expenditure costs, and remain easily accessible” for patient use [4]. Some platform features enable patients to self-measure blood glucose levels, log diet and healthy eating habits, track physical activities, enhance medication adherence, monitor insulin dosages, and receive real-time feedback on critical monitoring elements of a regimen management plan prescribed by a physician [1]. Condition management apps are on the rise and “now account for 40% of all digital health apps” with notable focus on disease-specific management apps [5]. The 5 areas of focus are chronic conditions that include mental health, diabetes, heart and circulatory conditions, nervous system disorders, and musculoskeletal conditions, 2 of which are leading causes of death in the United States (Table 1) [5]. A study by The IQVIA Institute for Human Data Science further reported that “clinical benefits across a broad array of conditions resulting from digital health application usage estimates a potential 1.4% savings in US national healthcare expenditures—equating to approximately $46 billion in total annual cost savings” (Figure 1) [5]. Currently, “there are over 300,000 health applications available in the market that address a variety of user needs from weight loss to management of chronic conditions, with diabetes being the most commonly targeted condition” [6].

**Table 1:** Disease-specific app by therapy area according to the IQVIA Institute for Human Data Science [5].

Therapy area	Proportion (%)
Mental health	28
Diabetes	16
Heart	11
Musculoskeletal system	7
Nervous system	7
Respiratory system	5
Cancer	5
Pain	4
Eyes and ears	4
Digestive system	4
Skin and tissue	3
Endocrine	3
Kidney disease	1
Hematology	1
Other	1

Within the last decade, mobile phone usage has substantially increased with approximately three-quarters of the US population owning mobile phones [7]. This has contributed to an increase in mobile access across patients with chronic conditions like diabetes. Mobile HIT tools and platforms use 4 main categories to promote adherence strategies for chronically ill patients: (1) SMS text messaging, (2) smartphone apps or software, (3) smartphones coupled with a wireless or Bluetooth compatible device, and (4) specific medical instruments connected to a smartphone via a cord [4]. Of these platforms, SMS text messaging has been reported to be the most commonly used by physicians and care providers, accounting for 40% of mobile HIT usage for appointment and medication reminders, patient education delivery, and the monitoring of symptoms through real-time data collection [4,8]. As HIT mobile solutions become more accessible for chronic disease patients, mHealth app designs aim to specifically accommodate this population.

### Mobile Management of Diabetes

A chronic disease is “defined broadly as a condition that lasts one year or more and requires ongoing medical attention, limits activities of daily living or both” [9]. The prevalence of chronic disease is continually rising in the United States and has moved far beyond the bar of epidemic concern, as 60% of adults in the United States have at least 1 chronic disease and 40% have 2 or more of these conditions [9]. Generally incurable but often treatable, chronic diseases contribute to the nation’s astounding $3.3 trillion annual health care costs and present heavy burdens on the nation’s health care spending [9]. Chronic diseases directly affect the economy, overall health care budgets, and employee productivity while also highlighting the urgent need for interdisciplinary solutions with long-term sustainability [10].

The top 3 chronic illnesses with the highest economic impact on United States health care systems are heart (cardiovascular) disease, cancer, and diabetes [9]. The cumulative aggregate of the 3 diseases constitutes a significant portion of the “nation’s annual health care expenditures” costing the “US health care system and employers $237 billion every year” in care and management [9,10]. Diabetes, or diabetes mellitus, is one of the leading causes of chronic disease deaths in America, with an estimated 30 million individuals diagnosed with the disease and another 80 million deemed to be prediabetic, which is the precursor to type 2 diabetes [9]. The disease is one of the most prevalent noncommunicable diseases, yet it is one of the costliest in terms of care and long-term management. The cost of long-term management of diabetes has contributed to heightened clinical interest in mobile, self-management solutions aimed at curtailing prolonged disease management costs and improving patient prognosis.

Diabetes presents in varying forms—type 1, type 2, and gestational—and disease development is attributed to insufficient production or use of insulin in the body leaving an excess of glucose in the bloodstream to be used as energy [11]. Type 1 diabetes is defined as an immune system attack on the cells that make insulin in the pancreas [11]. Alternatively, type 2 diabetes is typically diagnosed if the body does not sufficiently make or use insulin [11]. Both forms could present at any stage of life due to the body’s inability to manufacture or use insulin as intended [11]. Type 1 diabetes is most common among children, adolescents, and young adults but is treatable and manageable through the daily intake of insulin by those diagnosed with the disease [11,12]. Type 2 diabetes is most prevalent among adults and affects 90% of those with a diabetes diagnosis [12]. Gestational diabetes presents during pregnancy in women and is a fair indicator that type 2 diabetes might develop later in life if gestationally diagnosed while pregnant [11,12]. Healthy maintenance of blood glucose levels is key to diabetes management. The incorporation of modifiable lifestyle changes is often recommended to improve disease outcomes and offset future costs in long-term management for those with the type 2 disease [12].

### Diet

Diet is a major contributor of the leading forms of chronic disease in the United States and is often the primary risk factor of focus when chronic disease management is addressed. Many studies reiterate the direct correlation between poor dietary habits and chronic disease, yet 93.3 million US adults are obese and “about half of all-American adults—117 million individuals—have one or more preventable chronic diseases; many of which are related to poor quality eating patterns and physical inactivity” [13,14]. mHealth technologies have been used in recent years to track dietary glucose levels. In diabetes patients, the implementation of food tracking technologies has contributed to fewer occurrences of glycemic episodes leading to diabetic coma [15]. Mobile apps proven to assist in glucose monitoring are One Drop and DiabetesConnect, which are available from Apple iOS and Android app stores. These apps are used to track dietary intake but are not free of charge [16]. The One Drop app can sync data with insulin pumps, is Bluetooth accessible, and allows for access to support groups to strengthen disease management outreach and outcomes [16]. DiabetesConnect provides the user with access to downloadable and printable activity reports to be shared with their health care provider to enhance the clinical management experience [16]. Many diabetic individuals have also been found to employ the use of mHealth apps to assist with efficient tracking of blood glucose dietary intake, which is a prime clinical concern of a diabetes management regimen [16].

### Exercise

Another vital lifestyle factor that significantly influences the risk of developing type 2 diabetes is exercise [17]. Research consistently indicates that “physical inactivity is a primary cause to a myriad of chronic conditions/diseases” [18] and that “physical activity improves glycemic control and reduces the risk of cardiovascular disease and mortality in patients with type 2 diabetes” [17]. As a result, cardiovascular fitness is essential for the successful management of blood glucose levels, and research has shown that physical activity helps “reduce the risk of type 2 diabetes by approximately 50%” [17]. The Apple iOS Health App is an option for patients looking to track all exercise activities, including activity duration, stationary activities, measurements, and energy. With the availability of mHealth technology tools such as the Apple iOS Health App, awareness of physical activity levels can be effectively monitored and tailored to meet prescribed exercise regimens. The future points to mHealth apps that can “use embedded technology to showcase advanced uses of a smartphone to help in the prevention and management of chronic disorders such as type 2 diabetes” [19]. Indeed, the ability to monitor physical activity from handheld health devices or mHealth apps strengthens the capacity of patient disease self-management.

### Prescription Medication

An effective diabetes management program rests heavily on prescription medication adherence in addition to healthy diet and exercise. In fact, it is so crucial to diabetic management and control that it could prove fatal for patients who do not comply. “Direct health care costs associated with nonadherence” to prescription medication treatment plans “have grown to approximately $100-$300 billion of US health care dollars spent annually” [8]. The American Association of Clinical Endocrinologists /American College of Endocrinology and the American Diabetes Association support a “stepwise, progressive approach to pharmacotherapy” [20] in diabetes management. This specific type of pharmacotherapy refers to glycemic control, which is commonly associated with the use of metformin, a medication that inhibits the production of glucose in the blood [20]. With the introduction of monitoring health apps, such as Glucose Buddy Tracker, medications can be administered in a timely manner at the onset of irregular blood glucose levels [21] to prevent emergency room visits caused by delayed response. The Glucose Buddy Tracker app provides a monitoring log with alerts to check blood sugar levels in addition to prescription reminders and A_1C_ results [21]. To enhance management plans, these logs retain numerical and medication data that are transmitted to a primary physician of choice at no cost [21]. Lifestyle modification plans have become vital in the fight against rising chronic disease incidences, and the use of quality mHealth apps have shown favorable results towards increasing patient empowerment in the self-management of chronic diseases such as diabetes.

## Methods

A 2-fold approach was adopted involving the Apple iOS store and the Google search engine. A primary search for mobile diabetes apps was conducted on an Apple iPad (Apple Inc) within the Apple iOS store using the search term “diabetes apps.” The search rendered 179 results across the educational, recipe, and health and fitness sectors, most of which were excluded from this study. We identified and explored the top 5 most frequently used apps for features key to diabetes management: blood glucose A_1C_, insulin, physical activity, and prescription medication, along with app cost and other descriptive details. The most frequently used apps were considered to be those that had the highest number of consumer app reviews and the highest app rating on a 1 to 5-star Likert scale. The rationale behind searching within the Apple iOS store was entirely dependent on the iOS share (the percentage of people using iOS). A careful analysis of the market share and percentage of the population in the United States that use iOS revealed that approximately 62% use iOS and 38% use Android platforms; therefore, this study focused on data available in the Apple iOS store [22]. Although future research can expand to include other mobile operating system platforms, the intent of the present study was to capture patterns in mobile diabetes app use from a majority standpoint. The comments section under Table 2 captures the integration requirements and iOS-based apps.

A subsequent Google search was conducted using the search term “best Apple diabetes apps” to assess whether the selected Apple iOS apps would appear as the most frequently used across other online diabetes sources. We explored the first 3 diabetes search results—Healthline, Everyday Health, and Diabetes Apps–American Diabetes Association—and examined the 5 most frequently used apps across these sources against the same Apple iOS review criterion. In total, 12 unique apps that appeared across sources were examined. A blend of iOS users and Google search was deemed fit for this study owing to the percentage of people accessing or using these platforms to search for diabetes apps. The details of the app features are summarized in Table 2. Finally, a literature search was conducted using PubMed and National Center for Biotechnology Information (NCBI) databases, as well as Centers for Disease Control and Prevention (CDC), JMIR, and mHealth sources, to identify the means of furthering the efficacy and impact of mHealth apps in the self-management of diabetes.

## Results

The 5 most frequently used Apple iOS diabetes apps with the highest consumer ratings were OneTouch Reveal, Glucose Buddy Diabetes Tracker, Glucose Blood Sugar Tracker, One Drop, and Dario Blood Glucose Tracker (#1-#5, respectively, Table 2). All 5 mHealth apps provide feature capabilities that track blood glucose, and only 4 (#1-#4) include the additional ability to monitor A_1C_ and insulin levels. Two of the five apps (#4 and #5) enable physical activity tracking without the need to integrate with other health apps. Three of these mobile apps (#2-#4) provide prescription medication management abilities. Although significantly lower in customer reviews, the One Drop app was the only inclusive app to incorporate diabetes self-management capabilities for all assessed variances. Additional feature details such as cost, clinical recognition, data usability, integration compatibilities, and language were also recorded.

OneTouch Reveal had the highest number of consumer reviews at 39,000 within the Apple iOS store and received a rating of 4.7 stars out of 5.0 on likeability, offering only blood glucose, A_1C_, and insulin tracking features. This app is free of cost, available in 13 languages, and has physician summary report capabilities for 14, 30, or 90 days. It requires integration with Apple Health for physical activity tracking. The Glucose Buddy Diabetes Tracker has been descriptively ranked as the #1 diabetes app for more than the 10 years within the Apple iOS store but ranked second in consumer reviews with 13,000. Despite its US $3.99 app fee or US $14.99 to US $59.99 premium subscription options, consumers granted the app a higher rating than the OneTouch Reveal1 app, giving it a 4.8-star rating for disease management abilities. The Glucose Buddy Diabetes Tracker features support blood glucose, A_1C_, insulin, and prescription medication tracking while also requiring integration with Apple Health or other comparable fitness apps to track physical activities. This app is available in 30 languages and enables tracked data to be exported into PDF or Microsoft Excel files for physician evaluation purposes.

The One Drop app singularly offers collective tracking abilities of blood glucose, A_1C_, insulin, physical activity, and prescription medication within the app. Being free, available in 10 languages, and recognized across 15 peer-reviewed studies, One Drop only generated 10,000 consumer reviews and a lower rating of 4.5 stars in comparison to the other apps reviewed. Apple iOS users appeared less interested in the Dario Blood Glucose Tracker despite being the only app to meet the US Food and Drug Administration accuracy guidance standards, as it only brought in 8400 consumer reviews among its users compared to the 10,000 or more for the other 4 apps reviewed. This app did, however, receive the highest usability rating, receiving 4.9 stars out of 5.0 for its blood glucose, insulin, and nonintegration requirement in tracking physical activities. It is also free of charge, available in 30 languages, and enables on-demand data sharing of the monitored outcomes to be used for medical purposes.

The subsequent Google search for the “best Apple diabetes apps” returned Healthline, Everyday Health, and American Diabetes Association as the first 3 online source options. Healthline and Everyday Health are not scientific-based sources but are part of the Healthline Media brand whose health-related websites “reach more than 81 million people in the Unites States every month” [23]. The assessment of Apple’s iOS frequently used apps across other diabetes sources indicated the strong need to establish regulatory standards of mHealth apps. The Glucose Buddy Diabetes Tracker was the only app among the 3 online sources to appear as one of the leading Apple iOS apps for diabetes self-management. The app ranked first in frequent use within the Everyday Health and American Diabetes Association sources but second within Healthline and the Apple iOS store. The OneTouch Reveal app also had a shift in ratings, appearing first and highest in consumer reviews in the iOS store and Healthline online source. Other apps worth mentioning due to having chief diabetes tracking features include mySugar Diabetes Tracker Log (3500 reviews), Diabetes:M (518 reviews), Glooko (312 reviews), and Health2Sync (126 reviews).

**Table 2:** List of the top 5 diabetes self-management mobile apps by source.

App name by source			Rating		Number of reviewers		Price (US $)		Functions (a^a^, b^b^, c^c^, d^d^, e^e^)		Comments
**Source** **1:** **Apple** **iOS** **store**											
	1. *One* *Touch* *Reveal*^f^	4.7		39,000		0		a,b,c,d		Apple Health integration needed	
	2. *Glucose* *Buddy* *Diabetes* *Tracker*	4.8		13,000		3.99^g^		a,b,c,d,e		Other fitness integration	
	3. Glucose Blood Sugar Tracker	4.7		11,000		0		a,b,c,d,e		Health kit integration	
	4. One Drop	4.5		10,000		0		a,b,c,d,e			
	5. Dario Blood Glucose Tracker	4.9		8400		0		a,c,d			
**Source** **2:** **Healthline–Best** **Diabetes Apps**											
	6. *One* *Touch* *Reveal*	4.7		39,000		0		a,b,c,d		Apple Health integration needed	
	7. *Glucose* *Buddy* *Diabetes* *Tracker*	4.8		13,000		3.99^g^		a,b,c,d,e		Other fitness integration needed	
	8. *mySugar-* *Diabetes* *Tracker* *Log*	4.7		3500		3.99^g^		a,b,c,d,e		Apple Health integration needed	
	9. *Diabetes:M*	4.6		518		0		a,b,c,d		Apple Health integration needed	
	10. *Health2Sync*	4.7		126		0		a,b,c,d,e		Apple Health integration needed	
**Source** **3:** **Everyday** **Health**											
	11. *Glucose* *Buddy* *Diabetes* *Tracker*	4.8		13,000		3.99^g^		a,b,c,d,e		Other fitness integration needed	
	12. *mySugar–Diabetes* *Tracker* *Log*	4.7		3500		0		a,b,c,d,e		Apple Health integration needed	
	13. Diabetes Tracker by MyNetDiary	4.6		802		9.99^g^		a,b,c,d,e		Optional other fitness integration needed	
	14. *Diabetes:M*	4.6		518		0		a,b,c,d		Apple Health integration needed	
	15**.** * Health2Sync*	4.7		126		0		a,b,d,e		Apple Health integration needed	
**Source** **4:** **Diabetes** **Apps–American** **Diabetes Association**											
	16. *Glucose* *BuddyDiabetes* *Tracker*	4.8		13,000		3.99^g^		a,b,c,d,e		Other fitness integration needed	
	17. *mySugar–Diabetes* *Tracker* *Log*	4.7		3500		0		a,b,c,d,e		Apple Health integration needed	
	18. Glooko	4.7		312		0		a,c,d,e		Apple Health integration needed	
	19. Diabetes Pal	3.3		25		0		a,b,c,d,e			
	20. OnTrack Diabetes	2.3		4		0		a,b			

^a^a: dietary blood glucose.

^b^b: dietary.

^c^c: insulin.

^d^d: physical activity.

^e^e: Rx medicine.

^f^App names in italics indicated the most popular apps by source.

^g^Subscription offered.

## Discussion

### Principal Findings

Many studies point to mHealth apps as crucial weapons in the battle to find sustainable solutions to long-term chronic disease management. Not only can the use of these compact health solutions increase disease self-management abilities among chronic sufferers but consistent use of quality mHealth apps can also positively impact disease care and treatment and mitigate emergency room visits leading to long-term hospital stays.

Features key to the existence of a well-rounded diabetes management program were included and assessed in this study: blood glucose A_1C_, insulin, physical activity, and prescription medication. All 5 Apple iOS apps had blood glucose tracking capabilities, while the presence of the other assessed features varied by app. The Glucose Buddy Diabetes Tracker was the only app among all 3 of the online sources to appear as one of the leading Apple iOS apps for diabetes self-management.

Instead of using clinical standards to evaluate the quality and capabilities of mHealth apps, we evaluated apps based on consumer rankings and popular use. The most frequently used apps were considered to be those with the highest number of consumer apps reviews and the highest app rating on a 1 to 5-point Likert scale. mHealth apps collect vital health data that can be manipulated in various forms and be shared with attending physicians to strengthen disease-management plans for diabetes and other chronic health conditions alike.

### Challenges and Limitations

Although there are reports of the positive impact associated with the use of diabetes-related mHealth apps, there are several challenges and limitations—namely, patient demographics, smartphone accessibility, and privacy concerns—that continue to hinder increased use among patients with chronic health issues. In addition, over the course of 1 year, mHealth app downloads dropped drastically from more than 35% in 2015 to roughly 7% in 2016 [24]. This drop can be attributed to hidden costs, high data entry burden, loss of interest, and security and privacy concerns [24]. Data input frequency and users’ adherence to the time lines (as mentioned in the app) are factors that continue to be a challenge in this space. Maintaining data integration among various input parameters to generate a unified view and monitor the user’s condition while ensuring a high level of security remains another challenge in this space. The confidentiality of the patient information and adherence to HIPAA (Health Insurance Portability and Accountability Act) guidelines where applicable is another challenge. There is a definite need for a governing body to oversee and regulate the data transmission methods when the information is shared over the web. Even if the information is shared with a primary care physician or a provider, critical security touch points and monitoring will enhance patient outcomes.

### Sociodemographics

When assessing patient demographics as it relates to diabetes management, patient’s age and race play a crucial role in deciding whether mHealth apps should be implemented to fit the patients’ overall needs. For instance, according to a 2015 study, individuals who were found more likely to use mHealth or iOS apps were younger, more educated, of Latino or Hispanic ethnicity, earning higher incomes, and classified as obese by their BMI [25]. When it comes to smartphone use, younger populations are often highly proficient with and adaptable to smartphones [21]. In contrast, older adults might find the use of app-based mobile technologies to be challenging for diabetes management [21]. Regarding race and its role in the adoption of mHealth apps, evidence has shown that people of minority racial or ethnic groups and those that have lower health literacy use mHealth apps less when compared to individuals of nonminority racial or ethnic groups and to individuals that report higher health literacy, respectively [26]. Higher educational level and annual income tend to affect the adoption of these apps given the technological demand required of the user [27]. Consequently, the need for educational assistance of those with lower education and income levels is strongly needed to promote the benefits of diabetes self-management.

### Cost

The cost of technology alone has proven to be another challenge to mobile app usability. In order to take advantage of mHealth apps in the self-management of chronic disease, patients must first have the financial means to purchase a smartphone in which access to these solutions is housed. The latest version of popular smartphones using the Apple iOS operating system without a wireless service contract can cost from US $500 to US $700 per device [21]. In addition to the cost of the device, some iOS apps have associated costs as reported in Table 2. Research has shown that apps requiring payment for use compared with free apps are more likely to integrate health-literate design strategies, such as using plain language, clearly labeling links to app features, and providing effortless consumer usability functions [28]. Patients considered to be of low socioeconomic status are more likely to have low health literacy [26]. The cost of paid apps may not be affordable for patients with low health literacy, restricting them by default to using free apps and struggling with their limited features.

### Privacy

Privacy has become a concern as indicated by numerous reports of recent security breaches in the hospital industry, by the potential lack of access to information during power failures, and by computer server malfunctions [29]. As of December 27, 2018, the Department of Health and Human Services Office for Civil Rights received notifications of 351 data breaches of 500 or more health care records, and this number continues to rise [23]. Health data security and privacy concerns play a huge role in the lack of patients’ acceptance of mHealth apps [24]. With app developers presenting such an array of app options to consumers, the safety and effectiveness of patient information cannot be ensured.

### Conclusions

Given the severity of the nation’s chronic health epidemics, continued efforts are extremely necessary to find innovative solutions for improving the cost efficiency and sustainability of chronic care. mHealth apps continue to provide invaluable health solutions towards strengthening patient empowerment through the use of mobile disease self-management platforms. Mobile apps such as Apple iOS One Touch Reveal and Glucose Buddy Diabetes Tracker were shown to be popular, in frequent use, and ranked highly among Apple consumers and other diabetes online sources. However, mobile app choices are ultimately based on the users’ preferences and needs for effective disease management.

As the prevalence of diabetes and other chronic disease becomes more widespread, research should continue to expound upon the uses of mHealth technologies as solutions that increase disease self-management and improve health outcomes for sufferers of chronic disease. Many studies have pointed to the benefits of mobile management and self-oversight of chronic disease care, but usability must actively capture the attention of the end user to be effective. At this time, there remains no clear evidence explaining why patient adherence has remained low in diabetic patients compared to other chronic diseases [30].

mHealth apps have shown promise in the management of chronic disease, but the recent surge in the development of these digital health solutions demonstrates a growing need for a governing body that is knowledgeable of HIT, clinicians, policy makers, and patients in order to better define standards for effective chronic disease management. Variability in app features, cost, and tracking abilities could be reduced by regulatory uniformity, thereby increasing both self-care participation and improving diabetes outcomes.

## Abbreviations

CDC: Centers for Disease Control and Prevention
HIPAA: Health Insurance Portability and Accountability Act
HIT: health information technology
mHealth: mobile health
NCBI: National Center for Biotechnology Information

## References

[1] Istepanian RSH, Al-Anzi Turki M (2018). m-Health interventions for diabetes remote monitoring and self management: clinical and compliance issues. Mhealth.

[2] Robbins R, Krebs P, Jagannathan R, Jean-Louis G, Duncan DT (2017). Health app use among US mobile phone ysers: analysis of trends by chronic disease status. JMIR Mhealth Uhealth.

[3] Lunde P, Nilsson BB, Bergland A, Kværner KJ, Bye A (2018). The effectiveness of smartphone apps for lifestyle improvement in noncommunicable diseases: systematic review and meta-analyses. J Med Internet Res.

[4] Di Torro R, Lama G (1974). [A case of nephroblastoma associated with the nephrotic syndrome]. Minerva Pediatr.

[5] Aitken M, Clancey B, Nass D (2017). The growing value of digital health evidence and impact on human health and the healthcare system. IQVIA Institute for Data Science.

[6] Jimenez G, Lum E, Car J (2019). Examining diabetes management apps recommended from a Google search: content analysis. JMIR Mhealth Uhealth.

[7] (2017). Record shares of Americans now own smartphones have home broadband. Pew Research Center.

[8] Neiman AB, Ruppar T, Ho M, Garber L, Weidle PJ, Hong Y, George MG, Thorpe PG (2017). CDC grand rounds: improving medication adherence for chronic disease management - innovations and opportunities. MMWR Morb Mortal Wkly Rep.

[9] (2021). About chronic diseases. Centers for Disease Control and Prevention.

[10] Schmidt H, Dawson A, Barrett DH, Ortmann LW, Saenz C, Reis A A, Bolan G (2016). Chronic disease prevention and health promotion. Public Health Ethics: Cases Spanning the Globe.

[11] What is diabetes?. National Institutes of Health.

[12] (2019). About diabetes. Centers for Disease Control and Prevention.

[13] Hales C, Carroll Margaret D, Fryar Cheryl D, Ogden Cynthia L (2017). Prevalence of obesity among adults and youth: United States, 2015-2016. NCHS Data Brief.

[14] (2015). 2015-2020 dietary guidelines. US Department of Health and Human Services.

[15] (2019). Monitoring your blood sugar. Centers for Disease Control and Prevention.

[16] Kerr D, Hoppe C, Axelrod C (2017). Smartphone apps for diabetes management. Summit Healthcare.

[17] Hamasaki H (2016). Daily physical activity and type 2 diabetes: A review. World J Diabetes.

[18] Booth F, Roberts Christian K, Laye Matthew J (2012). Lack of exercise is a major cause of chronic diseases. Compr Physiol.

[19] Muralidharan S, Ranjani H, Anjana R, Allender S, Mohan V (2017). Mobile health technology in the prevention and management of Type 2 diabetes. Indian J Endocr Metab.

[20] Thrasher J (2017). Pharmacologic management of type 2 diabetes mellitus: available therapies. Am J Med.

[21] Ristau R, Yang J, White Jr (2013). Evaluation and evolution of diabetes mobile applications: key factors for health care professionals seeking to guide patients. Diabetes Spectrum.

[22] (2021). Mobile operating system market share United States of America. statcounter GlobalStats.

[23] (2018). Largest healthcare data breaches of 2018. HIPAA Journal.

[24] Zhou L, Bao J, Watzlaf V, Parmanto B (2019). Barriers to and facilitators of the use of mobile health apps from a security perspective: mixed-methods study. JMIR Mhealth Uhealth.

[25] Krebs P, Duncan DT (2015). Health app use among US mobile phone owners: a national survey. JMIR Mhealth Uhealth.

[26] Lyles C, Ratanawongsa Neda, Bolen Shari D, Samal Lipika (2017). mHealth and health information technology tools for diverse patients with diabetes. J Diabetes Res.

[27] Feroz A, Kadir MM, Saleem S (2018). Health systems readiness for adopting mhealth interventions for addressing non-communicable diseases in low- and middle-income countries: a current debate. Glob Health Action.

[28] Caburnay CA, Graff K, Harris JK, McQueen A, Smith M, Fairchild M, Kreuter MW (2015). Evaluating diabetes mobile applications for health literate designs and functionality, 2014. Prev Chronic Dis.

[29] Klonoff David C, Kerr David (2018). Overcoming barriers to adoption of digital health tools for diabetes. J Diabetes Sci Technol.

[30] Adu M, Malabu Usman H, Malau-Aduli Aduli E O, Malau-Aduli Bunmi S (2018). Users' preferences and design recommendations to promote engagements with mobile apps for diabetes self-management: Multi-national perspectives. PLoS One.

